# Natural Antioxidant Anthocyanins—A Hidden Therapeutic Candidate in Metabolic Disorders with Major Focus in Neurodegeneration

**DOI:** 10.3390/nu11061195

**Published:** 2019-05-28

**Authors:** Rahat Ullah, Mehtab Khan, Shahid Ali Shah, Kamran Saeed, Myeong Ok Kim

**Affiliations:** 1 Division of Applied Life Science (BK 21), College of Natural Sciences, Gyeongsang National University, Jinju 52828, Korea; rahatullah1414@gnu.ac.kr (R.U.); mehtabneuro@gnu.ac.kr (M.K.); alishahshahid@yahoo.com (S.A.S.); kamran.biochem@gnu.ac.kr (K.S.); 2 Department of Chemistry, Sarhad University of Science Information Technology (SUIT), Peshawar Khyber Pakhtunkhwa 25000, Pakistan

**Keywords:** oxidative stress, metabolic syndrome, Alzheimer’s disease, Parkinson’s disease, anthocyanins, neuroprotection

## Abstract

All over the world, metabolic syndrome constitutes severe health problems. Multiple factors have been reported in the pathogenesis of metabolic syndrome. Metabolic disorders result in reactive oxygen species (ROS) induced oxidative stress, playing a vital role in the development and pathogenesis of major health issues, including neurological disorders Alzheimer’s disease (AD) Parkinson’s disease (PD). Considerable increasing evidence indicates the substantial contribution of ROS-induced oxidative stress in neurodegenerative diseases. An imbalanced metabolism results in a defective antioxidant defense system, free radicals causing inflammation, cellular apoptosis, and tissue damage. Due to the annual increase in financial and social burdens, in addition to the adverse effects associated with available synthetic agents, treatment diversion from synthetic to natural approaches has occurred. Antioxidants are now being considered as convincing therapeutic agents against various neurodegenerative disorders. Therefore, medicinal herbs and fruits currently receive substantially more attention as commercial sources of antioxidants. In this review, we argue that ROS-targeted therapeutic interventions with naturally occurring antioxidant flavonoid, anthocyanin, and anthocyanin-loaded nanoparticles might be the ultimate treatment against devastating illnesses. Furthermore, we elucidate the hidden potential of the neuroprotective role of anthocyanins and anthocyanin-loaded nanoparticles in AD and PD neuropathies, which lack sufficient attention compared with other polyphenols, despite their strong antioxidant potential. Moreover, we address the need for future research studies of native anthocyanins and nano-based-anthocyanins, which will be helpful in developing anthocyanin treatments as therapeutic mitochondrial antioxidant drug-like regimens to delay or prevent the progression of neurodegenerative diseases, such as AD and PD.

## 1. Introduction

### 1.1. Metabolic Syndrome and Oxidative Stress

Metabolic syndrome (MetS) is a collection of several risk factors of metabolic disorders that occur together, raising the risk of developing cardiovascular disease and other health problems including type 2 diabetes and stroke. Metabolic syndrome, or syndrome X, has been a hot debate in clinical and research settings and has led to mortality and morbidity worldwide. According to the Third Report of the National Cholesterol Education Program Adult Treatment Panel III, MetS is characterized by different combinations of three or more features, such as hyperglycemia, a low level of high-density lipoprotein (HDL) cholesterol (HDL-C), hypertriglyceridemia, abdominal obesity, and hypertension [1]. The diagnostic criteria for metabolic syndrome have been defined by the World Health Organization [2], the National Cholesterol Education Program’s (NCEP’s) Adult Treatment Panel (ATP) III guidelines [3], and another organization [4]. There is a substantial increase in the epidemic proportion of metabolic syndrome (MetS) due to complex interactions between environmental factors and genetic factors, unhealthy dietary habits and sedentary lifestyles [5]. Multiple factors have been reported in the pathogenesis of metabolic syndrome [6]. One candidate, oxidative stress, results in the attack of cellular macromolecules, such as proteins, lipids and nucleic acids, leading to cellular dysfunction [6]. In addition, nutritional stress (i.e., a high fat and high carbohydrate diet) also stimulates oxidative stress, as evident by protein carbonylation, increased lipid peroxidation products, reduced glutathione (GSH) levels, and a decreased antioxidant system. Thus, ROS act as a pathogenic milieu in the development of several chronic diseases, including carcinogenesis, diabetes, obesity, hypertension, cardiovascular diseases, and neurodegenerations. A synergistic interaction between oxidative stress and metabolic disorders (MetS) has emerged, the understanding of which can be helpful in the identification of molecular targets, novel biomarkers, and effective drug development for the prevention and therapy of these diseases.

### 1.2. Reactive Oxygen Species (ROS): Sources and Free Radical-Scavenging Mechanisms 

In the fields of biology and medicine, free radicals are known as ROS or reactive nitrogen species (RNS) [7]. ROS encompass a broad category of highly reactive substances that are ubiquitous and short-lived derivatives of oxygen metabolism produced as intermediate products of normal cellular metabolism. ROS can be broadly classified into free radicals and nonradical groups (powerful oxidants). Free radicals consist of unpaired electrons that have short lifetimes (milliseconds-nanoseconds) due to a high energy state and include superoxide O2(−) and hydroxyl radicals (OH−). In contrast, nonradical ROS are relatively stable powerful oxidants that consist of singlet oxygen (^1^O_2_), hypochlorous acid (HOCl), ozone (O_3_), hydrogen peroxide (H_2_O_2_), and chloramines (RNHCl) [8]. Similarly, reactive nitrogen species (RNS) that contain nitrogen atoms consist of radical compounds, including nitric oxide (NO), nitroxyl (NO–) anions, peroxynitrite (NO−3), and nonradical compounds, i.e., peroxynitrous acid (HNO_3_) and nitrous oxide (N_2_O) [9]. Among ROS (O_2_.−, −OH, H_2_O_2_), superoxide (O_2_.−) is highly reactive and a cytotoxic agent.

**Figure 1:**
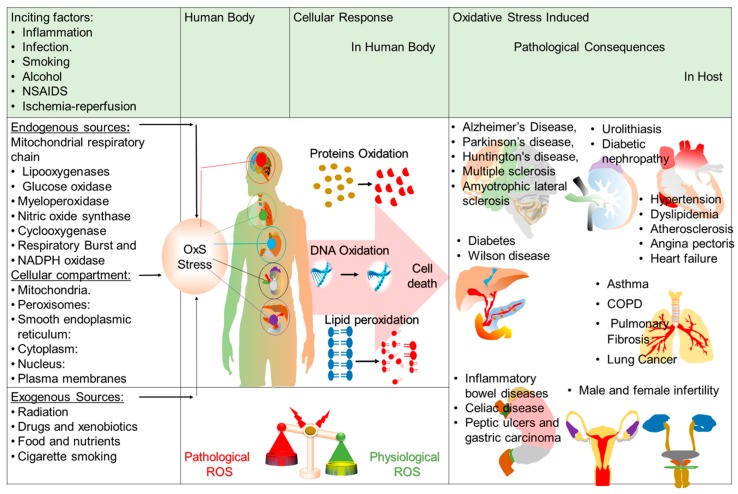
Schematic diagram showing various inciting factors, endogenous and exogenous sources leading to reactive oxygen species (ROS) generation in the human body followed by cellular response leading to pathological consequences in internal organs of the host. NSAIDs: Non-steroidal anti-inflammatory drugs, NADPH: Nicotinamide adenine dinucleotide phosphate, OxS: Oxidative stress, DNA: Deoxyribonucleic acid, COPD: Chronic Obstructive Pulmonary Disease.

Endogenous ROS sources comprise intracellular multicompartments (mitochondria, peroxisomes, endoplasmic reticulum, cytosol, nuclei, and plasma membranes), extracellular spaces and enzymes that catalyze ROS-generating chemical reactions (Figure 1) [8]. However, in most mammalian cells, the mitochondrial electron transport chain is the major site of ROS production [10]. In addition, exogenous or external ROS sources include multiple stimuli that accelerate oxidative stress, including tobacco smoke, air pollutants, ionizing and nonionizing radiation, xenobiotics, foods and drugs. Chemical agents, such as quinones [11], organic solvents, heavy metals (lead, chromium) and pesticides, are common exogenous sources of ROS [12]. ROS are also produced in response to ultraviolet (UV) radiation, alcohol consumption, cigarette smoking, ingestion of nonsteroidal anti-inflammatory drugs (NSAIDs), and many other exogenous agents. Ischemia-reperfusion (I/R) injury, infections, and various inflammatory processes also result in elevated levels of ROS [8]. Numerous exogenous and endogenous ROS sources are elucidated in detail in Figure 1. To cope with oxidative stress, human and animal cells have developed a ubiquitous antioxidant system. If the cellular antioxidant system is unable to overcome the amount of generated ROS, the excess ROS will deteriorate the normal cellular homeostasis leading to the oxidation of biological molecules [13]. The resultant oxidized or nitrated products of ROS will decrease biological activity, which leads to metabolic dysregulation and alterations in cell signaling [14] and thus accelerates the pathogenesis of various disease states [15,16]. 

An antioxidant may be defined as “a substance” that is present at a lower concentration than an oxidizable substrate and that has the ability to inhibit oxidation of the substrate [17]. For aerobic life, oxidation reactions are important, and abnormal ROS generation is harmful. ROS-induced oxidative stress can be prevented by several mechanisms, such as (i) preventative mechanisms, (ii) physical defense (iii) repair mechanisms, and (iv) antioxidant defenses [18]. Moreover, the body is equipped with antioxidants that act as a shield against the harmful effects of ROS and are collectively termed the antioxidant defense system [8]. This defense system consists of both enzymatic and nonenzymatic mechanisms. The endogenous enzymatic antioxidants include superoxide dismutase (SOD), catalase (CAT), glutathione peroxidase (GPX), glutathione reductase, superoxide reductases and heme oxygenase (HO-1) [19]. In contrast, endogenous nonenzymatic antioxidants include glutathione, thioredoxin, and melatonin. Similarly, exogenous antioxidants include vitamin C, vitamin E, carotenoid, minerals, and polyphenols, including flavonoids [8]. Taken together, collectively these findings indicate, that an imbalance between ROS levels and the antioxidant defense system contribute to the structural and functional loss of biological tissues in the onset and progression of numerous diseases. 

## 2. Oxidative Stress and Human Disorders

Accumulating lines of evidence confirm/suggest that oxidative stress plays a vital role in the development and pathogenesis of major health problems leading to cardiovascular diseases [20], joint disorders [21], eye disorders [22], lung and kidney disorders [23,24], atherosclerosis [25], liver and pancreatic diseases [26,27], aging [28], cancer [29], infertility [30], and neurological diseases (Alzheimer’s disease, Parkinson’s disease and amyotrophic lateral sclerosis) [31]. Herein, we have only focused on oxidative stress in AD and PD, and the previously described disorders are beyond the scope of this review. 

## 3. Oxidative Stress and Brain

The brain consumes approximately 20% of the total amount of the oxygen supply in the body, of which a significant portion is converted to ROS [32]. Evidence from AD or PD brain tissue samples supported elevated oxidative stress signs induced by ROS [33]. Oxidative stress damages all classes of major biomolecules (proteins, lipids, and DNA), and vulnerable neuronal population loss exceeding more than 90% results in neuronal dysfunction [34]. 

### 3.1. Oxidative Stress and Alzheimer’s disease (AD)

Alzheimer’s disease (AD) is a progressive, late-onset dementia and age-dependent neurodegenerative disorder characterized by an intracellular accumulation of tangles (insoluble hyperphosphorylated tau proteins) and extracellular plaques Aβ peptide deposition, i.e., (Aβ1–40 and Aβ1–42) [35]. The pathogenesis of AD has been considered a multifactorial origin [36], and its exact mechanism remains a question of debate. Various factors have been implicated in the etiology of the disease; oxidative stress is among one of the widely accepted factors [37]. Numerous studies have reported that oxidative stress is an early response in both the human AD brain [38] and animal models of AD, where it accelerates Aβ accumulation [39], suggesting a central role of oxidative stress in the early episode of AD pathogenesis [40]. Similarly, reactive nitrogen species (RNS) have also been found to contribute to AD pathology by aberrantly activating N-methyl-D-aspartate receptors (NMDARs) by glutamate (excitotoxicity) [7]. 

#### 3.1.1. Sources of Oxidative Stress in AD

**Figure 2:**
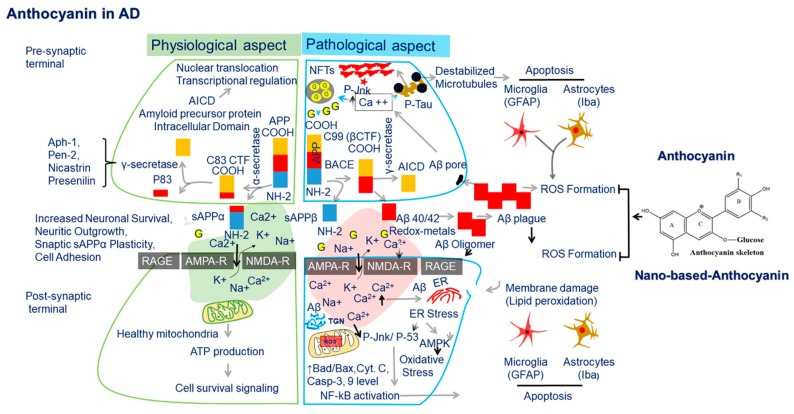
The schematic representation of physiological and pathological aspects (major sources of oxidative stress) in Alzheimer’s disease (AD) and their corresponding antioxidant defense approach by anthocyanin in cholinergic neurons. Mitochondrial abnormalities, advanced glycation end products (RAGE), redox-active metals (iron, copper and zinc), amyloid beta (Aβ) deposition, and alterations in cell signaling pathways are major sources of Oxidative stress (OxS) in AD. In an amyloidogenic pathway, the generated neurotoxic Aβ40/42 peptides from integral membrane protein Amyloid precursor Protein (APP) after sequential cleavage by β and γ-secretase is aggregated and formed oligomers before plaque (extracellularly) and neurofibrillary tangles (intracellularly) formation. This results in the disruption of calcium homeostasis, blocked ion channels, mitochondrial dysfunction impairment of energy metabolism, and glial cell activation that ultimately leads to oxidative stress resulting in neuronal cell death. In contrast, in a non-amyloidogenic pathway, APP is initially cleaved by α-secretase followed by further cleavage by γ-secretase excluding the possibility of Aβ generation and thus ROS production. Anthocyanin inhibited generated oxidative stress (OxS) in AD providing a neuro-protective effect. Abbreviations. BACE1, beta-site APP cleaving enzyme 1; βCTF, Beta site intracellular carboxyl terminal fragment; AICD, amyloid intracellular domain; APP, Amyloid precursor protein, GFAP, Glial fibrillary acidic protein, Iba-1, ionized calcium adaptor binding molecule 1, NF-κB, Nuclear factor-κB, p- JNK, phospo c-Jun N-terminal Kinase.

In AD, in addition to metabolically derived ROS, there are a number of additional contributory sources that play an important role in disease progression. The major sources of oxidative stress include mitochondrial, Aβ, glial cell, advanced glycation end product (AGE) altered cellular signaling, and metal abnormalities (Figure 2).

##### Mitochondrial Abnormalities 

The mitochondria and its role in the decision of cell fate to live or die is of substantial importance [41]. Evidence suggests that mitochondria have a dominant role in ROS production [42]. In AD, mitochondrial abnormalities/damage due to the deficiency of several key enzymes, including α-ketoglutarate dehydrogenase complex (KGDHC) and pyruvate dehydrogenase complex (PDHC), lead to ROS production [43,44]. Oxidative stress damages mitochondria and thus causes mitochondrial dysfunction, which ultimately induces apoptotic cell death followed by neurodegeneration [45]. In AD, the ranking of factors that contribute to mitochondrial dysfunction includes (i) low vascular blood flow to the brain due to chronic hypoxia/hypoperfusion [46], (ii) enhanced sporadic mutations in mtDNA [47], and (iii) a direct Aβ accumulation that occurs in mitochondria where it is processed in combination with amyloid-β protein precursor (AβPP) [48,49]. Reddy et al. also reported Aβ induced mitochondrial dysfunction in AD [50]. Furthermore, hyperhomocysteinemia, homocysteine [51,52] and apolipoprotein E4 (ApoE4) [53] are strong factors involved in mitochondrial dysfunction leading to AD development. 

##### Glial Cell Activation 

In the AD brain, senile plaques and neurofibrillary tangles (NFT) or injured neurons provoke inflammation in a similar fashion as inflammation that occurs in damaged peripheral cells or tissues. Aβ deposition activates glial cells [54], which express a wide range of inflammatory mediators, including cyclooxygenase, chemokines, and cytokines [55]. Studies have demonstrated a direct link between activated NADPH oxidase of microglia and Aβ peptide, resulting in the burst release of superoxide radicals and the enhanced production of hydrogen peroxide [56,57]. Nitric oxide (NO) produced by activated astrocytes and microglia reacts with superoxide to form peroxynitrite, thus leaving nitrotyrosine, i.e., an identifiable marker. The excess production of nitric oxide (NO) in AD can be measured from the increased amount of nitrotyrosine-modified proteins [58]. In the AD brain, amyloid plaques are surrounded by activated astrocytes with a high iNOS level [59,60]. In contrast, activated microglia involve the enzyme myeloperoxidase (MPO) in ROS production [61]. Myeloperoxidase (MPO) has an active role in the formation of nitrotyrosine-modified protein formation [62] and advanced glycation end product modifications [63], both of which are evident in AD. 

##### Advanced Glycation End Products 

In the Alzheimer’s disease (AD) brain, the accumulation of advanced glycation end products (AGEs) has been reported and is considered a feature of aging [64]. AGEs undergo redox cycling and play a vital role in ROS production in the presence of transition metals, thus promoting neuronal dysfunction in Alzheimer’s disease [65,66]. Similarly, other convincing evidence also supports the prominent role of AGEs and amyloid-β in ROS production by acting via RAGE, i.e., receptor for advance glycation end product [67].

##### Redox-Active Metals: Iron, Copper, and Zinc

The brain has a relatively high concentration of biometals (iron, copper, and zinc). Studies have confirmed that abnormal homeostatic mechanisms of copper, iron, and zinc accelerate redox-metal binding to Aβ and tau proteins in AD [68] via the Fenton reaction. For example, iron-induced-oxidative stress pledges various apoptotic signaling pathways, leading to synaptic dysfunction and neuronal cell death [69]. Similarly, copper binds with Aβ to form Aβ: Cu complexes, as well as its inappropriate binding with tau protein, trigger oxidative stress [70,71,72]. Moreover, zinc is hypothesized to be involved in APP processing [73], leading to greater Aβ deposition [74]. Similarly, zinc binding to tau protein promotes its aggregation and thus accelerates tau-related neuropathology in AD [75]. 

##### Amyloid beta (Aβ) Deposition 

Many researchers highlight the central role of Aβ-induced-oxidative stress in the pathogenesis of the Alzheimer’s disease (AD) brain [76,77]. In the amyloidogenic pathway, APP is first proteolytically cleaved by β-secretase (BACE1, beta-site APP cleaving enzyme 1), which results in the extracellular soluble sAPPβ (ectodomain) and an intracellular carboxyl terminal fragment (βCTF). The latter is then further cleaved by γ-secretase inside the cell membrane, releasing Aβ peptides (neurotoxic) and AICD, i.e., amyloid intracellular domain. However, in the non-amyloidogenic pathway, APP is first cleaved by α-secretase, resulting in soluble sAPPα (neuroprotective roles in synaptic plasticity and neuronal survival) and αCTF, i.e., membrane bound. The membrane bound αCTF is further cleaved by γ-secretase, releasing P83 peptide (shorter fragment) and AICD. Therefore, it excludes the possibility of Aβ generation [78]. Studies have reported that Aβ1–42 is more toxic than Aβ1–40, which play a vital role in hydrogen peroxide or ROS production [79]. Oxidative damage stimulates β-secretase and thus elevates Aβ1–42 levels [80,81]. Similarly, Aβ deposition has also been found in mitochondrial dysfunction in AD [82]. Aβ is also involved in cell membrane perforation by enhancing the calcium influx in cultured hippocampal neurons [83]. The increase in calcium influx triggers presynaptic glutamate neurotransmitter release, which activates postsynaptic N-Methyl-D-aspartic acid (NMDA) receptors [78] (Figure 2).

Upon the binding of excessive glutamate to N-methyl-D-aspartate (NMDA) receptors, i.e., ionotropic receptors, these receptors become permeable to calcium, potassium, and sodium ions. The abnormal intracellular Ca+2 levels induce mitochondrial damage and ROS production by increasing the mitochondrial Ca+2 burden [84]. Most importantly, Shahid et al. [85] reported that exogenously administered glutamate enhanced ROS production by activating AMPK and increasing the intracellular calcium level and expression levels of apoptotic and proinflammatory markers, such as caspase-3, COX2 and p-NF-kB, in both in vivo and in vitro models of glutamate-induced oxidative stress; this production is abrogated by anthocyanins. 

A relationship between Aβ oligomers and oxidative stress via NMDA receptor overactivation has been reported [86]. Aβ oligomers are responsible for an abnormal calcium influx, the activation of calpain, and degradation of dynamin 1 via NMDA receptors [87]. Taken together, this evidence suggests that the dysregulation of NMDA-R function induced by Aβ oligomers caused mitochondrial dysfunction followed by excessive ROS formation [86]. Moreover, Aβ has been reported as directly producing hydrogen peroxide via metal ion reduction [70]. Similarly, an increased level of 8-hydroxy-2-deoxyguanosine (8-OHdG), a marker of DNA oxidation, has been well documented in AD [88]. Overall, these effects of Aβ alter cellular function, resulting in neurotoxicity.

##### Alterations in Cell Signaling Pathways 

It is quite clear that oxidative stress induces alterations in the expression and activities of enzymes interacting within multiple signaling pathways. Among these pathways, stress-activated protein kinase (SAPK) pathways are the central mediators that play a vital role in amplifying stress signals into the nucleus. Therefore, in AD, oxidative stress signaling events cause JNK/SAPK activation and precede amyloid deposition [89]. Similarly, studies from multiple research groups have reported that Aβ induces a two- to three-fold increase in the activation of JNK/SAPK in multiple neuronal cell lines [90,91,92]. This activation of the JNK/SAPK pathway causes lipid peroxidation [93] and alterations of antioxidant enzymes, i.e., HO-1 and SOD1, in AD [94,95], and it upregulates BACE-1 expression, followed by elevated Aβ peptide levels, synaptic dysfunction, and neuronal cell death in AD [96,97]. Similarly, both in vitro and in vivo studies have reported stress-activated protein kinase (SAPK/JNK-P) in tauopathies [98]. Similar to reports on JNK/c-Jun, other studies highlight that oxidative stress increases the expression of Jnk/p38 levels and its strong association with senile plaque neurites and NFT in the pathogenesis of AD [89,99], as shown in (Figure 2). 

### 3.2. Oxidative Stress and Parkinson’s Disease

Parkinson’s disease (PD) is a chronic, progressive and the second most common neurological disease characterized by a loss of dopaminergic neurons (DA) in the substantia pars compacta (SNpc) and intracellular inclusion bodies (α-synuclein) [100]. The exact etiology of PD remains unclear, and understanding the precise mechanisms is the subject of intensive research. 

#### 3.2.1. Sources of Oxidative Stress in PD 

**Figure 3:**
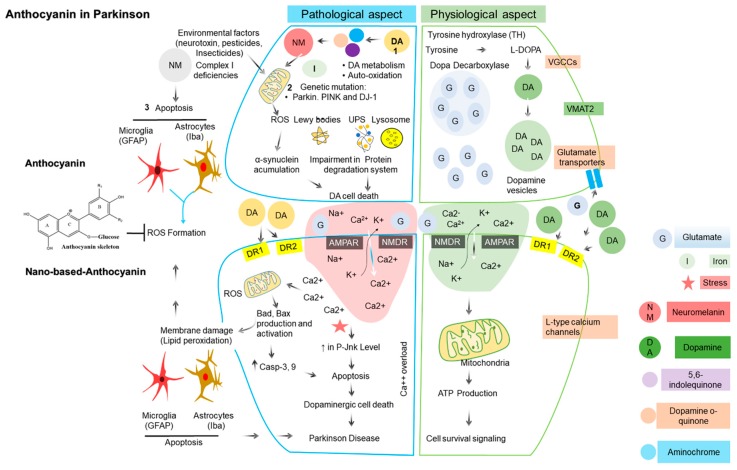
The schematic representation of physiological and pathological aspect (major sources of oxidative stress) in Parkinson’s disease (PD) and their corresponding antioxidant defense approach by anthocyanin in dopaminergic neurons. Dopamine metabolism, mitochondrial dysfunction, and neuro-inflammation are major sources of oxidative stress (OxS) in PD. Under the physiological state, release of dopamine (neurotransmitter) and glutamate (after synthesis and storage in their respective synaptic vesicles) are released into the synaptic cleft from presynaptic neuron results in physiological signaling in the postsynaptic neuron by stimulating their respective receptors. In contrast, under the pathophysiological state, oxidative stress (OxS) resulted either from dopamine metabolism, mitochondrial dysfunction and neuro-inflammation from dopaminergic neurons. Anthocyanin combats neuronal oxidative stress (OxS) from the aforementioned sources providing a neuro-protective effect in PD. Abbreviations: UPS, ubiquitin proteasome system; α-syn, α-synuclein; AMPA, α-amino-3-hydroxy-5-methyl-4-isoxazolepropionate; NMDA, N-methyl-D-aspartate; DR1 and DR2 (dopamine receptor 1 and 2); DA, dopamine, DJ-1, Protein deglycase.

There are a number of additional contributory sources that play an important role in the disease progression. However, the major sources of oxidative stress include dopamine metabolism, mitochondrial dysfunction, and neuroinflammation (Figure 3). 

##### Dopamine Metabolism 

In a normal physiological state, dopamine is released from presynaptic neurons into the synaptic cleft that activates dopaminergic receptors (D1 and D2) located on postsynaptic dopaminergic neurons. Dopamine (DA) is synthesized from tyrosine by tyrosine hydroxylase (TH) and is stored in synaptic vesicles, where its concentration is maintained in the physiological range. However, in the pathological state, when the cytosolic DA level is increased outside of the synaptic vesicle, e.g., metabolism or by autooxidation, it produces quinones, i.e., o-quinone, 5,6-indolequinone, and aminochrome [101]. DA quinones cause alterations in PD-related proteins, such as α-synuclein (α-syn), parkin (E3 ubiquitin protein ligase), DJ-1 (PARK7), superoxide dismutase-2 (SOD2), and UCH-L1 (ubiquitin carboxyl-terminal hydrolase isozyme L1) [101,102,103], which result in the inactivation of TH enzyme [104] causing brain mitochondrial dysfunction [105,106]. Similarly, under iron overload conditions, these quinones convert into neuromelanin involved in ROS production and [107] also exacerbate the neuroinflammatory process by microglial activation in PD [101]. In summary, dopamine itself directly or indirectly via its metabolites (quinones) and neuromelanin enhances mitochondrial ROS production (oxidative stress) or microglial activation (neuroinflammation), which ultimately leads to dopaminergic neuronal death in PD. 

##### Mitochondrial Dysfunction 

In PD, mitochondrial dysfunction is closely linked to increased ROS production [108]. Complex I deficiencies or its inhibition have been considered primary sources of ROS production in PD [109]. For example, rotenone and 1-methyl-4-phenyl-1,2,3,6-tetrahydropyridine (MPTP) are two well-known mitochondrial complex I inhibitors that have been reported in DA neuronal cytotoxicity [110]. The toxicity of rotenone has been documented to cause protein oxidation and Lewy body-like formation [111]. Blesa and Przedborski reported the mechanism by which 1-methyl-4-phenyl-1,2,3,6-tetrahydropyridine (MPTP) crosses the blood brain barrier and is oxidized to 1-methyl-4-phenylpyridinium (MPP+), where it accumulates in mitochondria [110]. This compound inhibits complex I in the mitochondrial electron transporter chain (METC) by disrupting electron flow in the mitochondrial electron transporter chain (METC), resulting in decreased ATP production and increased ROS generation [112]. Accumulating lines of evidence have linked genetic mutations of several mitochondrial proteins (α-syn, DJ-1, or PINK or parkin) and the promotion of oxidative stress, mitochondrial dysfunction, and DA cell death [113,114]. Similarly, multiple lines of evidence also support α-syn accumulation (a key constituent of Lewy pathology) inhibited mitochondrial complex I activity leading to ROS-mediated-DA neuronal death in both familial and sporadic PD [115,116]. Overall, complex I deficiencies or its inhibition, protein misfolding, genetic mutations in mitochondrial proteins and α-synuclein accumulation directly or indirectly affect mitochondrial function and thus play a vital role in the deleterious events involved in PD. 

##### Neuroinflammation

Barcia et al. [117] reported that neuronal loss is linked with chronic neuroinflammation via microglia-resident macrophages in the central nervous system (CNS) in PD. Evidence supports that environmental toxins (neurotoxins, insecticides, or pesticides), endogenous proteins [118], and Parkinson’s disease-associated proteins, such as α-syn, parkin, DJ-1, and LRRK2 [119], activate microglia that contribute to the neuroinflammatory response by initiating redox signaling in microglia [120]. DA oxidation leads to neuromelanin (NM) formation. Neuromelanin (NM) is released from extracellular dying neuromelanin (NM)-containing dopaminergic neurons in the SNpc. This process is followed by microglial activation, which results in ROS production and chronic neuroinflammation in PD [121,122]. Taken together, these inciting factors are directly or indirectly involved in microglial activation, which contributes to neuroinflammation in PD.

## 4. Medications for AD and PD 

### 4.1. Synthetic Approaches (Drugs)

The etiology of both AD and PD is of a multifactorial origin; therefore, the treatment of these diseases is also multitargeted (Table 1 and Table 2). Synthetic approaches have been reported that have high costs [123,124], drug-drug interactions, drug-food interactions, and severe side effects [125]. Therefore, to reduce the annual exponential increase in these neurological disorders and overcome their financial and social burdens, neuroscientists are continuously searching for alternative strategies to meet the desired clinical endpoints of affected individuals. That a diet rich in colorful fruits and vegetables reduces the risk of chronic illness is a well-accepted fact, emphasizing the interrelationship between health and diet. The old adage “an apple a day keeps the doctor away” sounds great considering healthcare costs and diet-related chronic illness. Notably, to maintain an active and enjoyable lifestyle as they age, consumers are seeking foods both to prevent disease and to achieve physical and mental health. Such foods provide consumers beneficial physiological effects beyond the required energy and essential nutrients [126]. Due to their health promoting-bioactive compounds, low cost, easy availability, and tolerability as well as offering a broad range of biological activities, including antidementia and cognitive-enhancing effects, fruits and vegetables containing natural antioxidants are currently considered an attractive research subject. 

### 4.2. Natural Approaches (Antioxidants) 

Epidemiological studies have reported antioxidant compounds with a broad range of biological activities. Antioxidants may be exogenous (natural or synthetic) or endogenous compounds. The natural antioxidant system includes enzymatic (catalase, glutathione peroxidase, and superoxide dismutase (SOD)) and direct-acting nonenzymatic (ascorbic acid, polyphenols, lipoic acid, and carotenoids) compounds and indirectly acting antioxidants that form adducts with metals and thus reverse ROS generation [31]. Natural antioxidants, including polyphenolic compounds obtained from dietary sources, such as anthocyanins from berries, catechins, and theaflavins from tea, curcumin from Curcuma longa or turmeric, resveratrol from grapes and peanuts have received substantial attention in the last few decades due to their broad range of biological activities [160,161,162]. 

## 5. Anthocyanins: A Natural Antioxidant

**Figure 4:**
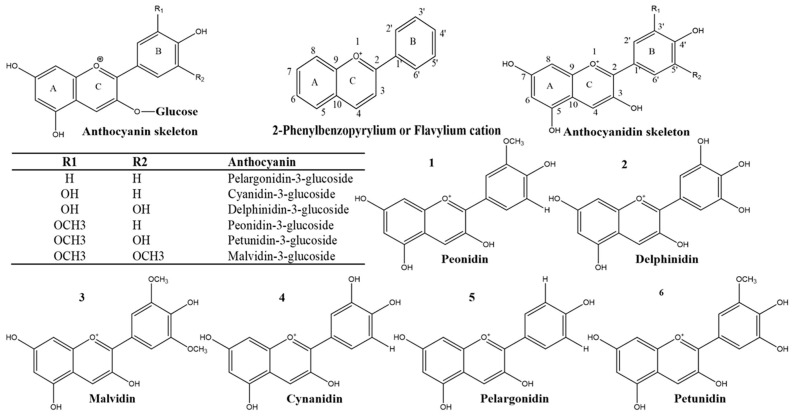
Chemical structures of anthocyanins (glycosylated with glucose), anthocyanidins and 2-phenyl benzopyrylium moiety or flavylium cations: two benzoyl rings (A and B) separated by a heterocyclic (C) ring. (**1–6**) most common anthocyanidins found in nature; cyanidin (cy), delphinidin (dp) pelargonidin (pl), peonidin (peo), petunidin (pt), and malvidin (mv).

Anthocyanins (a Greek word; anthos means flower and kyanos means blue) are an especially interesting and vigorously studied naturally occurring plant compounds that belong to one of the six subgroups of widespread plant constituents known as flavonoids [163]. The unique ability of anthocyanins to form flavylium cations makes them distinguishable from other flavonoids (Figure 4). The major sources of anthocyanins are blueberries, cherries, strawberries, raspberries, purple grapes, black currants, and red wine [164]. Anthocyanins have gained intensive research interest as a preventive and therapeutic plant agent. Historically, anthocyanin-rich food has been used as traditional medicine [126]. Anthocyanins and anthocyanin-rich extracts have diverse health benefits, leading to their widespread use in certain countries [126,163]. Thus, the old adage may be soon changed to “Binge on berries every day to keep the doctor away”. Nevertheless, research studies lack sufficient attention paid to anthocyanin phytochemicals compared to other flavonoids. Therefore, the purpose of this review is to highlight the antioxidant role of anthocyanins and their hidden potential in metabolic disorders, with a special focus on neurodegeneration (AD and PD); antioxidant therapeutic entities alone or in combination are discussed with the aim of overcoming the hazardous effects of current therapies for neurodegenerative disorders in the near future.

### 5.1. Chemistry, Structure-Activity Relationship and Mode of Action 

In plants, the anthocyanin molecule occurs as a glycoside that contains sugar (glycone moiety, i.e., mostly glucose, or xylose, rhamnose, galactose, or arabinose) and a nonsugar component (aglycone, i.e., anthocyanidin). In anthocyanins, the sugar component is esterified at position 3, which is missing in anthocyanidin in the same position (Figure 4). Chemically, anthocyanins are glycosylated, polymethoxy or polyhydroxy derivatives of 2-phenyl benzopyrylium moiety and are typically represented as flavylium cations: Two benzoyl rings (A and B) separated by a heterocyclic (C) ring. 

To date, over 700 structurally distinct anthocyanins and 27 known anthocyanidin molecules have been identified in nature. The most common anthocyanidins, which account for more than 90% of the known types, include cyanidins (cy, 50%), pelargonidins (pl, 12%), delphinidins (dp, 12%), petunidins (pt, 7%), peonidins (peo, 7%), and malvidins (mv, 12%) (Figure 4) [126,165]. The glycosylation of the glycone moiety (D-glucose, D-galactose, L-rhamnose, D-xylose, and arabinose) at the C-3 position is most common compared to other carbon positions, i.e., C-3/5/7/3′/4′/5′, where they are often acylated with aliphatic acids or by cyclic acids [166]. 

The structure-activity relationship substantially reflects the antioxidant potential of anthocyanins as it is not necessary that all of them possess similar efficacy for scavenging diverse ROS and RNS [167]. Anthocyanin-antioxidant activity is linked with numerous factors, including the number and position of the hydroxyl (-OH) and methoxy (-OCH3) groups in the basic anthocyanin skeleton, as well as the type of acylation and glycosylation pattern, e.g., the increased antioxidant effect of anthocyanins has a direct link with the number of the hydroxyl group in human leukemia cells. Similarly, anthocyanins that contain 3′,4′-dihydroxy groups are good metal ion chelators to form stable anthocyanin-metal complexes [166]. In addition to the degree and position of the -OH group in ring B, the position and degree of methoxy (-OCH_3_) also has a greater influence on the anthocyanin-antioxidant potential. For example, according to the results corroborated by Kähkonen and Heinonen (2003), the anthocyanins petunidin-3-glucoside and malvidin-3-glucoside showed a lower efficacy than delphinidin-3-glucoside and cyaniding-3-rutinoside [167]. Furthermore, anthocyanins’ antioxidant activity depends on the type of reactive species, i.e., the reactivity against the superoxide anion falls in the order of delphinidin cyanidin pelargonidin, whereas pelargonidin is highly effective against the hydroxyl radical [166]. 

Anthocyanins may act as extrinsic (direct) or intrinsic (indirect) antioxidants. The direct free radical scavenging effect is due to their potential to donate electron/hydrogen in their structure [166]. Anthocyanins have a high oxygen radical absorption capacity (ORAC) value, which may partially account for the neuroprotective effect [168,169]. Similarly, in in vitro models of H2O2 injury and Aβ peptide-induced toxicity, anthocyanins directly scavenge intracellular ROS formation [166,170]. Moreover, the indirect intrinsic antioxidant activity of anthocyanins may be due to (i) the restoration or increase in endogenous antioxidant enzymatic activities (SOD, CAT, and GPx) [171,172,173] resulting in higher antioxidant glutathione levels [174]; (ii) the activation of these endogenous antioxidants along with phase II detoxification genes by activating the antioxidant response element (ARE) via the redox-sensitive transcription factor NF-E2-related factor-2 (Nrf2) [175,176]; or (iii) the reduction in oxidative adduct formation in DNA and reduction in endogenous ROS formation by inhibiting xanthine oxidase and NADPH oxidase or by modifying arachidonic metabolism and mitochondrial respiration [165,177]. 

Bioavailability is defined as the quantity or fraction of the ingested dose of a drug or other substance that enters the systemic circulation [178]. It is widely believed that anthocyanins have a low bioavailability. However, recent investigations have shed new light on anthocyanin formulations that have higher bioavailability than those of previous studies. To overcome the bioavailability limitation, anthocyanin-loaded chitosan nanoparticles show a higher bioavailability and stability in the simulated gastrointestinal fluid than free anthocyanins [179,180]. Similarly, to increase the bioavailability and stability of anthocyanins due to their hydrophilic unstable nature, nanoparticle-based targeted drug delivery approaches using biodegradable nanoparticle formulations, e.g., PLGA@PEG nanoparticles in SH-SY5Y cell lines [181] and PEG-AuNPs in in vivo and in vitro AD models [182], have been used, improving the neurotherapeutic potential of anthocyanins. 

### 5.2. Pharmacological Activities of Anthocyanins

**Figure 5:**
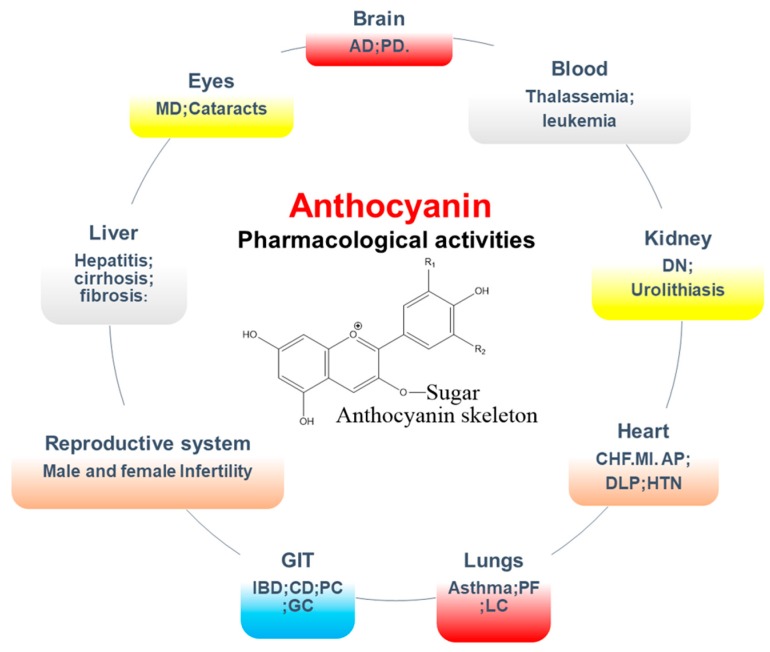
General overview of biological activities of anthocyanins. GIT: Gastrointestinal tract, IBD: inflammatory bowel disease, MD: Macular degeneration, HTN: Hypertension, DLP: Dyslipidemia.

Anthocyanins, anthocyanidins, and anthocyanin-rich extracts are of particular interest to nutritionists, due to their diverse health benefits, leading to the widespread use of these compounds in certain countries [126,163]. Based on multiple studies, including animal models, cell-line studies, and human clinical trials, it has been concluded that anthocyanins may play a vital role in numerous pathologies, including showing anticancer, antiviral, antidiabetic (via AMPK activation), anti-inflammatory (downregulation of COX-2 and iNOS), cardioprotective (enhancing SOD antioxidant activity), and neuroprotective activities (diminishing glutamate-induced neurotoxicity and increasing glutathione content) (Figure 5) [183,184]. The broad scientific evidence of the beneficial effects of these compounds on human health and their increasing popularity explains their availability in the form of nutraceuticals and food supplements [185]. 

## 6. Anthocyanins: A Neuroprotective Agent

**Figure 6:**
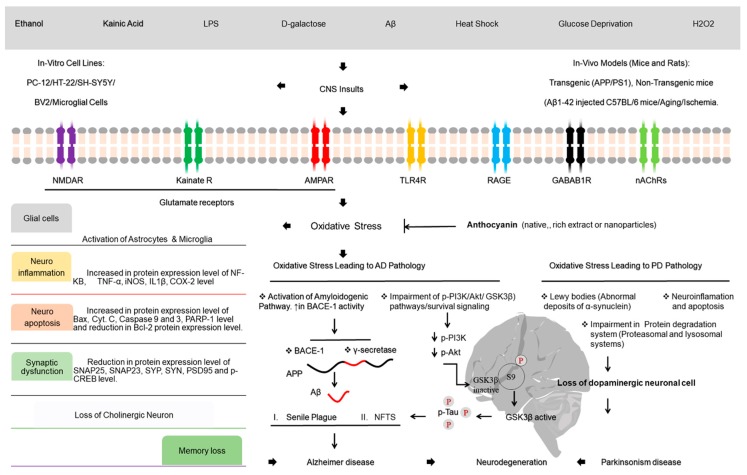
Neuroprotective effect of anthocyanin against oxidative stress by CNS insults (signaling) in AD and PD. p-Tau: phosphorylated Tau Protein.

It is known that the brain is the busiest organ and the most sensitive to oxidative stress [31]. Aging, a fundamental cause of brain dysfunction, is accompanied by long-term effects of oxidative stress [186]. Studies have reported the neuroprotective effect of several anthocyanins in AD and PD [187] involving multiple mechanisms, including antioxidant activities [188]. A previous study reported the in vivo antioxidant activity of an orally administered anthocyanin mixture (Vaccinium myrtillis L., 100 mg/kg bw) against psychological stress-induced cerebral oxidative stress and dopamine abnormalities after psychological stress was induced by cutting off whiskers in distressed mice [189]. Moreover, the strong free radical scavenging effect of purple sweet potato anthocyanins (PSPAs) has been reported against Aβ-induced-toxicity in PC12 cells [190]. Similarly, cyanidin-3-O-glucoside isolated from mulberry fruit has been reported with neuroprotection against cerebral ischemia [191]. Notably, in APP1-PS1 transgenic mice and in aged humans, the neuroprotective effect of anthocyanins from blueberry juice (12 weeks) and Concord grape juice has been well documented [192,193]. Due to the mounting evidence of the diverse biological activities of anthocyanins, our group [194] reported the neuroprotective effect of anthocyanins against oxidative stress induced by kainic-acid via the AMPK pathway in primary prenatal rat hippocampal neurons and a mouse hippocampal cell line (HT22 cell line) (Figure 6). Similarly, the authors of Reference [195] further explored the neuroprotective effect against ethanol-induced oxidative stress via the GABAB1 receptor intracellular signaling pathway in prenatal hippocampal neurons. The results indicated that anthocyanins significantly inhibited ethanol-induced inhibition of glutamatergic neurotransmission, GABAB1R activation, synaptic dysfunction, and neuronal apoptosis by upregulating the phosphatidylinositol-4,5-bisphosphate 3-kinase PI3K/Akt/ GSK3-beta pathway in the postnatal rat hippocampus brain (P7) and downregulating p-JNK, p-NF-kB, and COX-2 involved in apoptosis in the CA1, CA3 and DG regions of the hippocampus in the developing rat brain (Figure 6). Moreover, the authors of References [196,197] reported similar findings of an anthocyanin-neuroprotective effect against D-galactose and LPS-induced oxidative stress-mediated neurodegeneration in adult rat brains and adult mouse cortex. Furthermore, [198] evaluated the neuroprotective effects of anthocyanins in both in vitro (mouse hippocampal HT22 cells) and in vivo (APP/PS1 mice) mouse models of Alzheimer’s disease. More recently, Khan MS [199] extended similar studies and confirmed the antioxidant effect of anthocyanins in in vivo (8 week old C57BL/6N mice) and in vitro (HT22 cells and BV2 microglia cells). The results of histological, immunoblot and confocal analyses indicated that the consumption of anthocyanins prevented ROS production, neuroinflammation (by reducing p-NF-kB, IL-1β, and TNF-α), and neuronal apoptosis (as evident from the reduced expression of Bax, cleaved caspase-3, cytochrome c, and cleaved PARP-1 and increased expression of antiapoptotic Bcl-2 protein). Similarly, anthocyanins increased the levels of survival proteins (p-Akt, p-GSK3β) leading to improved hippocampus-dependent memory function (by upregulating memory-related protein levels) and thus preventing neurodegeneration (Figure 6).

Globally, Parkinson’s disease (PD) is the second most common age associated with disease after AD [200]. Evidence suggests that a polyphenol-rich diet provides neuroprotective properties in lowering the risk of PD [201]. Numerous phytochemicals, including green tea [202], curcumin [203], resveratrol and oxyresveratrol [204,205], have been found to be neuroprotective in cellular and animal models of PD [206]. Recent epidemiological studies have reported that the intake of berries, such as strawberries and blueberries, has a neuroprotective effect in PD due to the presence of polyphenols, including anthocyanins and proanthocyanidins [207]. Consistent with these findings, anthocyanin- and/or proanthocyanidin-rich extracts also alleviate rotenone induced neurotoxicity by reversing mitochondrial dysfunction in PD [208]. Overall, these observations suggest that anthocyanins act as a neuroprotective agent in lowering the risk of PD and/or slow disease progression.

### Nanoparticle Approach of Anthocyanins in Neuroprotection

Due to the presence of phenolic hydroxyl groups in their chemical structure, anthocyanins are unstable and easily oxidized into quinones, resulting in reduced bioavailability and the loss of biological activity. To overcome these problems and enhance their neuroprotective effect, F.U. Amin et al. [181] for the first time used a nanomedicine therapeutic approach and effectively encapsulated anthocyanins in a biodegradable nanoparticle formulation, i.e., poly(lactide-co-glycolide) (PLGA), using polyethylene glycol (PEG)-2000 as a stabilizing agent; the authors reported their neuroprotective effect via the p38/JNK pathway against Aβ1-42-induced oxidative stress in SH-SY5Y cell lines. This nano-based neuroprotective approach of anthocyanins was then further extended in an animal model of Alzheimer’s disease by T.Ali et al. [209] using polyethylene glycol-gold nanoparticles (PEG-AuNPs) in an Aβeta1-42 injected mouse model of Alzheimer’s disease. Interestingly, the authors found that anthocyanin-loaded PEG-AuNPs (PEG-AuNPs) were more effective than native anthocyanins in reducing the neuropathological disorder of Alzheimer’s disease via the PI3K/p-Akt/p-GSK3β pathway. Similarly, M. J. Kim et al. [188] further evaluated anthocyanin-loaded PEG-gold nanoparticles (PEG-AuNPs) in both in vitro and in vivo models of Alzheimer’s disease via the NF-KB /JNK/GSK3β signaling pathway against Aβ1-42-induced neurodegeneration. Consistent with previous findings, similar findings were obtained using anthocyanin-loaded PEG-AuNPs. Thus, the conjugation of anthocyanins with nanoparticles provides a novel approach that is a helpful, effective, important, and promising strategy in nanomedicine for the prevention of age-associated neurodegenerative diseases. Taken together, this broad impressive evidence/approach opens a future therapeutic window for the encapsulation of anthocyanins (PLGA @ PEG or PEG-AuNPs) and offers a novel, considerable, effective, and promising nanomedicine strategy to improve the efficiency of anthocyanins using nanodrug delivery systems in the field nanomedicine for the prevention AD and PD. 

## 7. Safety and Toxicological Aspects of Anthocyanins

Anthocyanins are the most widely consumed flavonoid constituents and their intake is nine-fold higher than that for dietary flavonoids [183]. Accordingly, the use of anthocyanins (E163) in beverages and foods in certain countries, including Japan and the USA, was approved by the Scientific Committee on Food in 1975 and the Joint Food and Agricultural Organization/World Health Organization Expert Committee on Food Additives (JECFA) in 1982 (EFSA, 2013) [210]. Toxicological Reports from the JECFA (1982) concluded that the intake of anthocyanin-rich-extracts is associated with low toxicity based on hematology, clinical parameters, and teratogenic and motility analyses [163,211].

## 8. Anthocyanins as Mitochondrial Drug-Like Antioxidants and Their Possible Combinations 

**Figure 7:**
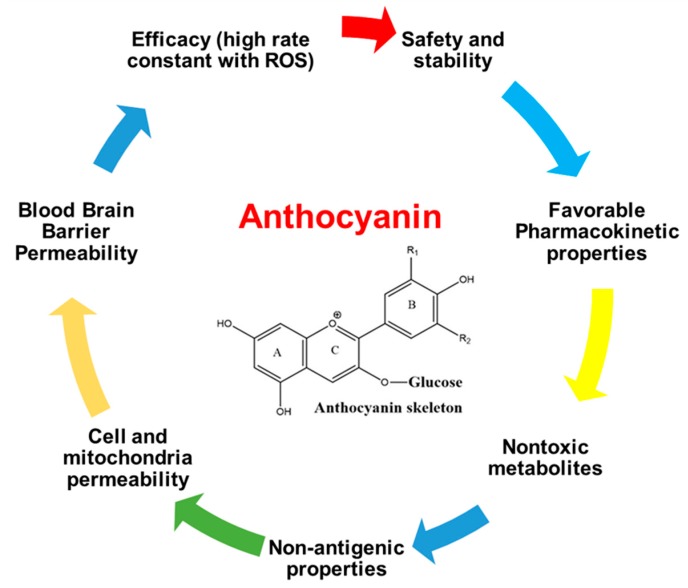
Desirable properties of anthocyanin as a mitochondrial drug-like antioxidant.

Considerable factors that require particular attention to develop anthocyanins as a mitochondrial drug-like antioxidant include efficacy, safety, stability, favorable pharmacokinetic properties, non-antigenic properties, cell and mitochondria permeability, and having nontoxic metabolites. Notably, anthocyanins as antioxidant molecules must cross the blood–brain barrier (BBB) and preferentially show better oral bioavailability in AD and PD as summarized in Figure 7. 

Studies have supported antioxidant supplementation alone or in combination with cholinesterase inhibitors to improve the cognitive function in patients with neurological disorders [7]. The antioxidant activity of anthocyanins may be enhanced by other phytochemicals or vitamins that are also abundant in fruits. These compounds may interact with each other synergistically [212]. For example, common dietary flavanols undergo synergistic antioxidant effects with common anthocyanins. M. Rossetto et al. [213] demonstrated a synergistic antioxidant effect of catechin and malvidin 3-glucoside in linoleic acid peroxidation induced by free radicals in micelles. Similarly, the lessons learned from previously synthesized and studied hybrid drugs (acetylcholine esterase inhibitor; tacrine) in combination with antioxidants (melatonin, coumarin, vitamin E) provide hope for a possible therapeutic combination of anthocyanins with a class of anti-AD drugs (Table 1) in the near future [7]. 

## 9. Antioxidants Under Clinical Trials

To date, more than 635 anthocyanins have been identified in nature [183], among which 26 clinical trials are available (16 completed) that strongly suggest future clinical trials [163]. In human clinical trials, numerous antioxidants are under investigation for their neuroprotective effects. Federally funded Alzheimer’s disease centers (an unpublished trial) previously reported that the intake of vitamin E (2000 units/day) and/or selegiline (deprenyl, a MAO-B inhibitor; 10 mg/day) has a slowing effect on AD progression [214]. Similarly, vitamin E, curcumin, and melatonin were found with encouraging results in the reduction of both Aβ tauopathies in in vitro and in vivo AD models [125]. In 2003, Kanowski et al. [215] reported the therapeutic effect of ginkgo biloba special extract EGb 761 (240 mg/day) in improving cognitive function in AD patients with dementia. Similar confirmatory findings were subsequently reported using diets (containing vitamin E alone or in combination with vitamin C or alpha-tocopherol) or antioxidants (Salvia officinalis/Melissa officinalis) in mild to moderate AD patients [125]. 

Likewise, use of huperzine A in China, and the US initiated its Phase II clinical trial after the joined efforts made by Alzheimer’s Disease Cooperative Study and NIH National Institute on Aging. Similarly, Phase II clinical trial of curcumin in AD patients is also instigated by John Douglas French Foundation Institute for the Study of Aging. In early PD patients, Stanley Fahn in 1991, reported an open trial of high-dose antioxidants including vitamin E (DL-α- tocopherol) and ascorbate (vitamin C) for assessment of antioxidants in the brain resulting in slowness to PD progression. Recently, in North America, a large multicenter and controlled clinical trial of Deprenyl and Tocopherol Antioxidant Therapy of Parkinsonism (DATATOP) is ongoing for the assessment of an antioxidant (tocopherol; 2000 U/day) used in combination with MAO- B (deprenyl; 10 mg/day) in a total of 800 subjects with early PD. The outcome of this DATATOP study will provide vibrant information regarding the role of antioxidants in the treatment of PD [216]. 

Taken together, these initial clinical trials on antioxidant and supplements may eventually overlap synthetic treatment and would answer the remaining questions of antioxidants in a clinical trial for AD and PD patients in near future.

## 10. Conclusion and Future Perspectives

As neurological disorders are of multi-factorial origin, oxidative stress requires considerable attention as one of the confirmed components, making it a key therapeutic target in combating these neurological disorders. The recent challenges of adverse effects and failed clinical trials, due to a higher toxicity associated with available synthetic compounds for neurological treatments (AD PD), suggest switching to natural compounds as a possible alternative approach. Herein, we argue that targeting oxidative stress with anthocyanins /nano-anthocyanins in diet or supplements as a universal preventive and therapeutic natural-antioxidant drug entity, presents a promising entry point into the aforementioned neuropathies, rather than flooding the body with synthetic compounds that come with side effects.

A nanotechnology-based drug delivery system offering anthocyanin nanoparticles (PLGA@PEG/(PEG-AuNPs) and an encapsulation approach enhanced by pharmacokinetics, pharmacodynamics, and controlled-release profiles in addition to BBB permeability, targets intracellular signaling and, most importantly, ROS scavenging capabilities of anthocyanins in various cellular and animal models in the aforementioned neuropathies. Importantly, it is worth noting that valuable information and lessons learned from failed clinical trials urge the need for the development of desirable ‘drug-like’ properties and strong rationale that may serve as the future direction for antioxidant drug-development. Going forward, we believe that in-depth analyses and sustained future research efforts are necessary and will open a new therapeutic window for uncovering the dark side (underlying molecular mechanism) of anthocyanins or nano-based-anthocyanins in neuro-protection. Similarly, its synergistic combinations, exploration of composition/particular molecular type/distinct structures, pharmacokinetic, effective functional doses, and metabolite concentrations in the affected brain region are still missing and need to be answered by designing synthetic nano-anthocyanins as therapeutic mitochondrial medicine to delay or prevent the progression of oxidative stress mediated cognitive disorders (AD PD) before entering and meeting their desirable clinical endpoints. The new version of the old adage will soon become “Binge on berries, pop in purple potatoes, revel with radishes, grab the grapes, and wind down with red wine.” Enjoy!

## Figures and Tables

**Table 1:** Pharmacological Treatment of AD.

S. No	Group	Classification of AD Drugs	Mechanism of Action	Side Effect	Ref. No.
1	**Anticholinesterase** **Inhibitors (AChEI):** (Currently approved Drugs for AD).	Donepezil(Aricept), Galantamine (Razadyne), Rivastigmine (Exelon), Tacrine(Cognex).	Increased Ach level by Inhibiting AchE.	Nausea, Diarrhea, Vomiting, Loss of appetite, Abdominal pain, Increased Frequency of bowel movements, Bradycardia, Hepatotoxicity (Tacrine/withdrawn from market).	[116]
2	**NMDA Receptor Antagonist**	Memantine(Namenda)	Increased Ca++influx (overload) thus blocking glutamatergic overstimulation.	Headache, Constipation, Confusion, and Dizziness.	[127]
3	**Antihypertensive Drugs** -**Diuretics** -**Angiotensin -1 Receptor Blockers (ARB):** **-Angiotensin-Converting Enzyme Inhibitors (ACE-I):** **-Calcium Channel Blockers (CCB):** **Note.** Dihydropyridine are more effective than nondihydropyridine agents in reducing incidence of AD). **-Beta Blockers**	**Diuretics:** **A.Thiazide Diuretics:** i) Thiazides: -Hydrochlorohiazie (HCTZ or HCT) (Apo-Hydro) -Benzthiazide (Dytide). ii) Thiazides like: -Chlorthalidone(Thalitone), -Metolazone (Zaroxolyn), -Indapamide (Lozol). **B. Loop Diuretics:** -Furosemide (Lasix), -Ethacrynic acid (Edecrin). **C. Potassium-sparing Diuretics:** i) Aldosterone Antagonist: -Spironolactone(Aldactone) -Eplerenone (Inspra). ii) Na+ Channel Blocker: -Amiloride (Midamor) -Triamterene (Dyrenium). **D.Carbonic Anhydrase Inhibitors:** -Acetazolamide (Diamox), -Dorzolamide (Trusopt) **Osmotic diuresis:** (Mannitol, Glycerol, Isosorbide and urea). **ARB:** -Candesartan (Atacand), -Valsartan (Diovan), -Losartan (Cozaar). **ACE-I:(Centrally acting):** -Captopril(Capoten): -Ramipril (Altace), -Lisinopril(Zestril, Prinivil), -Fosinopril (Monopril). **ACE-I:(Non-Centrally acting):** -Benazepril(Lotensin), -Enalapril(Vasotec), -Quinapril (Accupril). **CCB:** **Dihydropyridine:** -Amlodipine(Norvasc) -Nifedipine (Adalat). -Benzothiazepines: -Diltiazem (Cardizem LA). **Phenylalkylamines:** -Verapamil (Isoptin). **BB:** -Propranolol(Inderal), -Carvedilol(Coreg), -Nebivolol(Bystolic).	**Diuretics:** Decreased CSF amyloid-beta (Aβ1-42) level due to Hypokalemia associated with diuretics. **ARB:-** Decreased Aβ production and increased Aβ degradation. **ACE-I:** (Controversial) -Increased K+-mediated acetylcholine release, -Degradation of Aβ, Antioxidant potential and vascular protective effects. **CCB:** -Decreased intracellular calcium level by inhibiting calcium channels. -Decreased Aβ production. **BB:** -Decreased γ secretase activity i.e., Aß production.	Hyponatremia, dizziness, thirst, muscle cramps Hypokalemia (except potassium sparing diuretics). Dizziness, headache, drowsiness, nausea, cough (low incidence than ACEi), hyperkalemia, muscle and bone pain. **ACE-I:** Dry cough (due to increase in bradykinin level), hyperkalemia and fatigue. **CCB:** Constipation, flushing, drowsiness, edema and drowsiness. **Beta Blockers:** Coldness of extremities (hands and feet), dry mouth, skin and eyes, diarrhea and constipation. **Others:** Congestive Heart Failure, Vivid dreams, Parasthesias, Depression, Bronchial Bronchospasm, Night mares and Sexual dysfunction.	[128,129,130,131,132,133]
4	**NSAIDs.** (Anti-inflammatory drugs)	**COX-2 Inhibitor:** -Celecoxib(Celebrex) -Rofecoxib(Vioxx) -Valdecoxib (Bextra) **COX-1 Inhibitor:** -Ibuprofen (Brufen) -Aspirin(acetylsalicylicacid) -Naproxen(Anaprox) -Flurbiprofen (Anazin)	-Decreased neuroinflammation.	Gastrointestinal and Renal toxicity.	[134]
5	**Secretase inhibitors**	**BACE inhibitors** -Verubecestat (MK-8931), -Lanabecestat (AZD3293 or LY3314814), -Elenbecestat (E2609). **Gamma secretase inhibitor:** -Semagacestat (LY450139)	-Decreased Aβ production.	-Skin disorders, Small-bowel obstruction, Paranoia, and Increased agitation	[135,136]
6	**Insulin**	**Insulin Rapid Acting:** -Insulin Lispro (Humalog) -Insulin Aspart (Novolog) -Insulin Glulisine (Apidra) **Short-Acting:** -Regular Human (Humulin R) **Intermediate-Acting:** -NPH Human (Humulin N) **Long Acting:** -Insulin Determir (Levemir) -Insulin Glargine (Lantus)	-Glucose homeostasis -increased Aβ clearance by enhancing IDE and α-secretase activity.	-Weight gain, Hypoglycemia, Local reactions (allergic reactions), Mitogenic properties.	[137,138]
7	**Cytokines**	-Etanercept (Enbrel R) (TNF-α modulator)	-Potent antagonist of TNF alpha.	-Headache, Stomach pain, Weakness and Cough.	[139]
8	**HuperzineA** (Lycopodium alkaloid)	-HuperzineA	-Potent inhibitor of AChE -Anti-oxidant -Anti-apoptotic -Anti-inflammatory.	-Nausea, Vomiting, Sweating, Blurred vision.	[140]
9	**Polyphenols**	-Curcumin, Resveratrol	-Anti-inflammatory -Anti-oxidant -Aβ clearance –Chelating agent.	-Diarrhea, rash, Headache and yellow stool	[127,141]
10	**Herbal supplements**	-Ginkgobiloba -Panax gingseng -Withania somnifera	**Ginkgobiloba:** Inhibition of mitochondria dependent apoptosis OR Aβ aggregation inhibition. **Panax gingseng:** Reduction in Aβ peptide level. **Withania Somnifera:** Inhibition of AChE, reduction in of Aβ level, Antioxidant and anti-inflammatory activity.	-Nausea, Vomiting, Restlessness.	[142,143,144]
11	**Hormones**	-Melatonin/Estrogen (Estrace)	**Melatonin:** Anti-oxidant, Antiifibrillogenic and Attenuates Aβ toxicity **Estrogen:** Increased growth, survival and cholinergic activity, antioxidant and enhanced non-amyloidogenic metabolism of APP.	-Melatonin: -Depression, Headache, Sleepiness and irritability. -Estrogen: Weight gain and Post-menopausal breast cancer.	[145,146,147]
12	**MAO-Is:** (MAO-Ai and MAO-Bi)	**MAO-Ai and MAO-Bi (non-selective):** -Phenelzine (Nardil), -Tranylcypromine (Parnate), -Isocarboxazid (Marplan). **MAO-Ai (Selective):** -Clorgyline (irreversible), -Moclobemide (reversible). **MAO-Bi (Selective):** -Selegiline(Deprenyl)	-Accelerates nonamyloidogenic pathway. -Inhibited Aβ and tau pathophysiology. -Increased different neurotransmitters level in brain.	Significant Weight gain, Severe Orthostatic hypotension, Hypertensive Sexual Dysfunction.	[148,149]
13	**Lipid-lowering drugs**	**Statins:** -Lovastatin (Mevacor), -Simvastatin (Zocor), -Rosuvastatin (Crestor), **Fibrates:** -Clofibrate (Atromid-S), -Gemfibrozil (Lopid) **Others:** -Ezetimbe, torcetrapib, -implitapide. niacin	**Statins:** Inhibit HMG-CoA reductase ezyme, and thus Suppress cholesterol biosynthesis. **Fibrates:** Stimulate β-oxidation of fatty acids -Lowers plasma level of fatty acid and triacylglycerol.	Hepatotoxicity Carcinogenic Myopathy Nephrotoxicity, For-example, Cerivastatin (Removed from worldwide market due to serious myopathy/Rhabdomyolysis).	[150,151]
14	**AD Immunotherapy**		-Use of anti-Aβ protein antibodies (vaccine).		[127]

Abbreviations. IDE, Insulin Degrading Enzymes; TNF, Tumor Necrosis Factor; APP, Amyloid Precursor Protein; MAO-Ai and MAO-Bi, (Monoamine Oxidase A and B Inhibitors); HMG-CoA reductase, (3-hydroxy-3-methyl-glutaryl-coenzyme A reductase; AChE, Acetylcholinesterase; Ca++, Calcium; CSF, Cerebrospinal fluid; ARB, Angiotensin -1 Receptor Blockers; ACE-I, Angiotensin-Converting Enzyme Inhibitors; K+, Potassium NSAIDs, non-steroidal anti-inflammatory drugs; CCB, Calcium Channel Blockers; BB, Beta Blockers.

**Table 2:** Pharmacological Treatment of PD.

S. No.	Classification	Drugs	Mechanism of Action	Side Effect	Ref. No.
1.	**Dopamine agonists**	**Ergot derived dopamine agonists:** -Bromocriptine (Parlodel) -Pergolide (Permax) -Cabergoline (Dostinex) **Non-Ergot derived dopamine agonists:** -Pramipexole (Mirapex) -Ropinirole (Requip)	The antiparkinsonian effects of Dopamine agonists (DA) is due to their direct activation of dopaminergic receptor (D1 and D2).	**Ergot-derived-dopamine agonists:** Hallucination, Delusions, orthostatic, hypotension, exacerbation of dyskinesias, skin inflammation, erythromelalgia, digital vasospasm, paraesthesias and pleural effusion or pulmonary infiltrates. **Non-Ergot-derived dopamine agonists:** Nausea, Hypotension, Confusion and somnolence and Exacerbation of dyskinesias.	[152,153].
2.	**COMT inhibitors**	**Entacapone** (Comtan®), **Tolcapone** (Tasmar®).	-Inhibition of COMT enzyme in periphery, thus reducing levodopa degradation into 3-O methyldopa and increased levodopa availability in brain where it is converted into dopamine.	Tolcapone (Tasmar^®^) (black box warning), Fulminant liver failure, vivid dreams, diarrhea, urine discoloration(orange), drowsiness, visualhallucinations and dyskinesia.	[154,155]
3.	**DOPA decarboxylase inhibitor (DDCi)**	Levodopa (L-DOPA), Carbidopa (Lodosyn), Or Combination of both.	-Inhibition of DOPA decarboxylase in periphery, that caused levodopa degradation into dopamine (unable to cross BBB), and thus enable exogenously administered DOPA/levodopa to reach in brain in sufficient quantities, where it is converted to dopamine.	Motor fluctuations, Dyskinesias (“peak-dose dyskinesias”, “biphasic dyskinesias” and “wearing-off” dyskinesias	[153,156]
4.	**MAO-B Inhibitor**	Selegiline(Eldepryl, Emsam, Zelapar)-Rasagiline (Azilect) -Safinamide (Xadago), -Tranylcypromine (Parnat, non-selective)	These drugs work by inactivating MAO enzyme that caused inactivation of dopamine neurotransmitter.	High blood pressure, nausea, constipation Headache, difficulty falling asleep, blurred vision, cheese reaction (tranylcypromine).	[157]
5.	**Anticholinergic drugs**	Trihexyphenidyl (Artane) Benztropine (Cogentin) Procyclidine (Kemadrin) Biperiden (Akineton)	Reduction of persistent tremor or ameliorate dystonia or dyskinesias associated with DOPA decarboxylase inhibitor, or reduction in extrapyramidal side effects induced by antipsychotic agents.	Anticholinergic side effects: Dry mouth, Constipation, Urinary retention, Bowel obstruction	[158]
6.	**Antiviral drug**	Amantadine (Symmetrel)	-Antagonists of NMDA receptor as well as dopamine re-uptake inhibitor, therefore increase extracellular dopamine levels.	Depression, anxiety, hallucination, dry mouth and constipation.	[159]

Abbreviations. D1 and D2, Dopaminergic receptors; COMT, Catechol-O-methyltransferase; L-DOPA, levodopa or L-3, 4-dihydroxyphenylalanine; MAO, Monoamine oxidases; NMDAR, N-Methyl-D-aspartate receptor; DOPA decarboxylase inhibitor (DDCi).
